# FcRn Antagonism Leads to a Decrease of Desmoglein-Specific B Cells: Secondary Analysis of a Phase 2 Study of Efgartigimod in Pemphigus Vulgaris and Pemphigus Foliaceus

**DOI:** 10.3389/fimmu.2022.863095

**Published:** 2022-05-18

**Authors:** Maud Maho-Vaillant, Magdalena Sips, Marie-Laure Golinski, Gestur Vidarsson, Matthias Goebeler, Johanna Stoevesandt, Zsuzsanna Bata-Csörgő, Bianca Balbino, Peter Verheesen, Pascal Joly, Michael Hertl, Sébastien Calbo

**Affiliations:** ^1^ Department of Dermatology, Rouen University Hospital, Rouen, France; ^2^ INSERM U1234, Normandie University, Rouen, France; ^3^ argenx, Ghent, Belgium; ^4^ Department of Experimental Immunohematology, Sanquin Research and Landsteiner Laboratory, Academic Medical Centre, University of Amsterdam, Amsterdam, Netherlands; ^5^ Department of Dermatology, Venereology and Allergology, University Hospital Würzburg, Würzburg, Germany; ^6^ Department of Dermatology and Allergology, University of Szeged, Szeged, Hungary; ^7^ Department of Dermatology and Allergology, Philipps-Universität Marburg, Marburg, Germany

**Keywords:** pemphigus vulgaris (PV), pemphigus foliaceus (PF), efgartigimod, immunoglobulin G, FcRn, B cells

## Abstract

**Background:**

Immunoglobulin G (IgG) levels are maintained by the IgG-recycling neonatal Fc-receptor (FcRn). Pemphigus vulgaris and pemphigus foliaceus are debilitating autoimmune disorders triggered by IgG autoantibodies against mucosal and epidermal desmogleins. Recently, a phase 2 clinical trial (NCT03334058; https://clinicaltrials.gov/NCT03334058) was completed in participants with pemphigus using efgartigimod, an FcRn inhibitor, in combination with prednisone. Efgartigimod demonstrated an early effect on diease activity and was well tolerated. In addition to the safety and efficacy assessment, clinical trials present an opportunity to gain more insights into the mechanism of disease, the mode of action of treatment, and potential for corticosteroid-sparing activity.

**Objective:**

The aim of our study was to assess the impact of FcRn antagonism by efgartigimod on immunological parameters known to be directly involved in pemphigus pathology, such as cellular and serological responses.

**Methods:**

We investigated total and antigen-specific IgG subclass level kinetics during and after treatment, assessed antigen-specific B-cell responses, followed T- and B-cell immunophenotypes, and analyzed how different immunophenotypes link to clinical response.

**Results:**

Treatment resulted in reduction of total IgG as well as autoreactive IgG antibody levels. Surprisingly, unlike total IgG and vaccine- or natural-infection-elicited IgG, which returned to baseline levels after stopping efgartigimod treatment, autoreactive antibody levels remained low in several study participants. Efgartigimod showed no effect on total leukocytes, neutrophils, monocytes, or lymphocytes in patients treated with extended efgartigimod therapy. Intriguingly, antigen-specific analyses revealed a loss of desmoglein-specific B cells in several participants responding to efgartigimod, in line with prolonged reduction of pathogenic IgG levels.

**Conclusions:**

Efgartigimod treatment of participants with pemphigus improved their conditions and exerted an immunomodulatory effect beyond the blockade of IgG recycling. Further studies in larger populations with an appropriate placebo control are needed to confirm these potentially important observations to establish long-term clinical responses in autoimmune diseases.

## Introduction

Autoimmune diseases increasingly affect the human population and present significant healthcare burden, particularly in Western societies (1). It is estimated that more than 2.5% of the population is affected by autoantibody-driven autoimmune diseases (2) where autoreactive antibodies are directly pathogenic.

The presence of autoantibodies can be reduced, in principle, by B cell-depleting therapies such as rituximab, which has proven effective in a number of immunoglobulin G (IgG)-mediated autoimmune disorders (3) including pemphigus (4, 5). However, the emergence and prolonged existence of autoantibodies in general is a result of complex immune signaling linked to breaking of tolerance, possibly due to normal immune response turning cross-reactive and thereby attacking host proteins. This process involves multiple cells and pathways (6), including T lymphocytes and innate immune cells and most likely affinity maturation, providing the means for the development of cross-reactivity. It is now recognized that dysfunctional T lymphocytes facilitate B-cell autoimmune responses (7, 8), and B cells play a reciprocal role in CD4+ T-cell activation (9, 10). For example, pathogenic IgG autoantibodies frequently show clear signs of affinity maturation suggestive of CD4+ T cell-driven autoimmune B-cell response (11). B cells, in turn, can stimulate T cells by acting as antigen-presenting cells (APCs) or by producing pro-inflammatory cytokines (9, 10, 12, 13). Once the autoimmune response with the production of pathogenic autoantibodies has fully developed, it can be efficiently self-maintained by continuous activation of autoreactive B cells leading to constant formation of short-lived plasma cells, long-lived plasma cells, or both (14).

IgG autoantibodies can be pathogenic by directly blocking molecular interactions in the hosts (Fab-mediated) and/or by Fc-mediated effector functions involving complement or Fc receptors (15) and by promoting activation of T cells (16). Autoantigen uptake and presentation by APCs, fueled by the autoantibodies themselves through Fc-receptors [classical FcγR (17) but also the neonatal Fc receptor (FcRn) (18)] and/or complement-mediated uptake (19), may also reinforce inflammation and autoantibody production. Various therapeutic approaches targeting disparate immune components are therefore extensively tested in autoimmune diseases including pemphigus (20).

Pemphigus has been attributed to autoantibodies targeting desmogleins (Dsg), a group of transmembrane desmosomal glycoproteins ensuring the structural integrity of the epidermis. Pemphigus vulgaris (PV) is primarily caused by Dsg-3-specific antibodies, while pemphigus foliaceus (PF) is caused by antibodies targeting Dsg-1 (21). Despite our increasing understanding of the molecular mechanism driving autoimmune diseases, broad systemic immunosuppression is still the predominant treatment, although it is associated with adverse events ranging from direct toxicity to increased risk of infection (22, 23).

Antagonizing the FcRn, a major histocompatibility complex class I-like molecule involved in the recycling of IgG, and thereby responsible for the long half-life of IgG (24, 25), has been explored as a strategy to treat IgG-mediated autoimmune diseases such as generalized myasthenia gravis (gMG) (26), immune thrombocytopenia (ITP) (27), or pemphigus (28). Efgartigimod is an Fc fragment derived from human IgG1 engineered for increased affinity for FcRn, thereby outcompeting endogenous IgG leading to its degradation (24). FcRn inhibition shows clinical efficacy that is linked to early removal of pathogenic IgG autoantibodies from the circulation. However, the effect of prolonged suppression of pathogenic IgG has not been extensively studied yet. Nevertheless, sustained suppression of IgG by efgartigimod for up to 34 weeks has been explored in participants with pemphigus in a phase 2 trial and proven to afford lasting clinical benefit while remaining well tolerated (28).

Results from the phase 2 trial of efgartigimod in participants with pemphigus have been previously reported (28). Briefly, disease control (DC; defined as no new lesions and established lesions starting to heal) was achieved in 28 of 31 (90%) participants after a median time of 17 days. In cohort 4, end of consolidation (EoC, defined as the time at which no new lesions have developed for a minimum of 2 weeks and the majority, i.e., approximately 80% of established lesions, have healed) was achieved in 11 of 15 (73%) participants after a median of 43 days. Complete clinical remission (CR; defined as the absence of new lesions and established lesions completely healed), measured only in cohorts 3 and 4, was achieved in 14 of 22 (64%) participants after a median of 92 days while receiving a median daily dose of 0.26 mg/kg of prednisone. Efgartigimod was well tolerated overall and most adverse events were mild to moderate in intensity. Total serum IgG as well as anti-Dsg-1/3 levels were suppressed during treatment. However, after treatment discontinuation, IgG levels rose to baseline while anti-Dsg-1/3 remained suppressed (70% reduction from baseline for anti-Dsg-1, and 42% reduction for anti-Dsg-3) (28). Therefore, we hypothesized that long-lasting removal of autoantibodies *via* FcRn inhibition may alter autoimmune signatures and restore immune homeostasis.

In order to further understand this phenomenon, we performed this secondary analysis of the phase 2 trial participants to investigate autoreactive and protective antibody titers as well as B-cell and T-cell phenotypes from peripheral blood of efgartigimod-treated participants.

## Methods

### Study Design and Treatment Intervention

Extended treatment with efgartigimod and concomitant low-dose prednisone was gradually introduced in an open-label, adaptive phase 2 trial in participants with mild to moderate PV or PF (ClinicalTrials.gov: NCT03334058). Seven participants (7 PV) were treated for 15 weeks in cohort 3 and 15 participants (8 PV, 7 PF) were treated up to 34 weeks in cohort 4 as reported earlier (28). PV or PF diagnosis had to be confirmed by positive direct immunofluorescence and positive indirect immunofluorescence and/or Dsg-1/3 ELISA. Participants were either newly diagnosed or relapsing, with mild to moderate disease severity (pemphigus disease area index [PDAI] <45 at baseline) (29). The treatment period was preceded by a screening period of up to 3 weeks and followed by a treatment-free follow-up period of 10 weeks. In cohort 3, intravenous (i.v.) efgartigimod was administered weekly at 10 mg/kg for 4 weeks as induction followed by administrations every other week for 12 weeks as maintenance period. In cohort 3, efgartigimod could be initiated as monotherapy or in combination with 20 mg/day of prednisone at the discretion of the investigator, and prednisone could be tapered starting in the maintenance period. In cohort 4, i.v. efgartigimod was dosed weekly at 25 mg/kg body weight until EoC, defined as the time at which no new lesions have developed for a minimum of 2 weeks and the majority, i.e., approximately 80% of established lesions have healed. Thereafter, participants were dosed every other week. In cohort 4, efgartigimod was initiated with concomitant prednisone (20 mg/day) in all newly diagnosed participants and relapsing participants off therapy, or at the tapered dose at which relapse occurred. The oral prednisone dose could be tapered as of EoC. No other systemic treatments for pemphigus were permitted during the study, but topical corticosteroids, analgesics, and supportive care for corticosteroid therapy (e.g., vitamin D, proton-pump inhibitors, and specific diets) were allowed (28).

### Ethics

The study was conducted in accordance with the Good Clinical Practice guidelines, in conformity with the ethical principles of the Declaration of Helsinki and was compliant with all relevant country-specific laws. The study protocol and all other appropriate study-related information were reviewed and approved by the ethics committees or institutional review boards of every participating center.

### Photography

Photography was conducted as routine practice for documenting dermatological conditions in medical practice. Additional participant consent was obtained for sharing anonymized participants’ pictures with the sponsor.

### Immunoglobulin Assays

Pharmacodynamic analysis included determination of serum levels of total IgG, IgG subclasses, and anti-Dsg-1 and -Dsg-3 autoantibodies by ELISA (Euroimmun, Germany). Serum levels of protective vaccine antibodies against tetanus toxoid (TT; Indirect EIA, Virotech), varicella zoster virus (VZV; CLIA, Diasorin), and pneumococcal capsular polysaccharide (PCP; EIA, The Binding Site Group) were measured for all participants. A customized Addressable Laser Bead ImmunoAssay (ALBIA) test was performed to determine the anti-Dsg-3 IgG subclasses at different time points. The ALBIA method used was previously described (30). Briefly, 20 μg of anti-HIS antibody (clone HIS.H8) was coupled to 1.25 × 10^6^ fluorescent Bio-Plex R COOH-microspheres (Bio-Rad, USA) with the Bio-PlexR amine coupling kit (Bio-Rad) according to the manufacturer’s protocol. After coupling, coated beads were either used immediately or stored at −20°C in the dark. Before use, 100 ng of recombinant Dsg-3 expressing a 6×His Tag were incubated with 1,000 beads for 15 min at room temperature (RT) and then washed. Immediately prior to their use, coated beads were vigorously agitated for 30 s. Then, 10 μl of beads (containing 1,000 beads) was added to 100 μl of participants’ or control serum diluted 1/100 in PBS with Ca^2+^ and Mg^2+^ supplemented with 1% FCS, in Bio-Plex Pro Flat bottom plates (Bio-Rad). Plates were incubated for 1 h 30 min at RT in the dark on a plate shaker at 850 rpm. Blank (no serum), negative controls (healthy donor serum), and positive controls (human anti-Dsg-3 positive serum) were included in every assay. Beads were collected with a magnetic washer (Bio-Rad) and washed three times with 150 μl of PBS 1× containing 0.1% Tween-20. Biotinylated mouse anti-human IgG-subclass specific secondary antibody (Southern Biotech, USA) was added (at 1/125 dilution for anti-IgG1 and for anti-IgG2, at 1/200 dilution for anti-IgG3 and for anti-IgG4) for 45 min at RT under shaking. After washing, beads were incubated with 50 μl of streptavidin-R-phycoerythrin at 1/400 dilution for 15 min. Finally, beads were resuspended in 100 μl of PBS and mean fluorescence intensity (MFI) was determined on a Bio-PlexR apparatus using the Bio-PlexR Manager Software 4.0 (Bio-Rad). The anti-Dsg-3 IgG subclass serum levels were determined with the following formula: (MFIserum/MFICalibrator) × 100, in which the calibrator was an anti-DSG3 positive control used on every analysis and set to 100 arbitrary units (AU). For determination of Dsg-3-reactive antibodies, the positivity threshold was set at the mean value obtained from 36 healthy donors plus 2 standard deviations. EUROIMMUN anti-Dsg-1 ELISA (Euroimmun, Germany) with modifications to the detection system was employed to identify anti-Dsg-1 IgG subclasses. Rabbit anti-human IgG HRP detection antibody was replaced by anti-human IgG1 or anti-human IgG4 biotinylated (Southern Biotech, USA), followed by streptavidin HRP used at 1/10,000 dilution to detect Dsg-1 specific IgG subclasses and used at 1/10,000 dilution. CIC-C1q EIA kit (A001, Quidel) was employed to detect levels of C1q-associated IgG aggregates (circulating immune complexes [CIC]) in sera of selected participants at different time points according to the manufacturer’s protocol. Calibrators provided by the kit were used to determine expression level.

### Isolation of Peripheral Blood Mononuclear Cells

A total of 60–80 ml of whole blood was collected into 10 BD Vacutainer CPT tubes, remixed immediately and centrifuged within 2 h of blood collection at room temperature to separate peripheral blood mononuclear cells (PBMCs) and red blood cells. Following centrifugation, PBMCs were resuspended into the plasma and transported to the analytical laboratory of the Department of Dermatology at the Philipps University Marburg, Germany, within 24 h. Upon sample receipt, tubes were subjected to PBMC purification, flow cytometry analysis, and cryopreservation of the remaining cells.

### Phenotypic Profiling by Multiparameter Flow Cytometry and Detection of Dsg-3-Specific B Cells

PBMCs were washed twice with PBS supplemented with 1% FCS and 1 × 10^6^ cells were stained for T and B lymphocyte subset analysis including detection of Dsg-3-specific B cells as previously described (13). The following antibodies were used for T lymphocyte panel analysis: mouse anti-human CD4 (RPA-T4, BioLegend), mouse anti-human CD45RA (HI100, BioLegend), mouse anti-human CXCR5 (J252D4, BioLegend), mouse anti-human CD25 (M-A251, BioLegend), mouse anti-human CD127 (A019D5, BioLegend), mouse anti-human CXCR3 (G025H7, BioLegend), and mouse anti-human CCR6 (G034E3, BioLegend). The gating strategy is shown in **Supplementary Figure 1A** for one representative participant. The following antibodies were used for B-cell panel analysis: mouse anti-human CD45 (2D1, BioLegend), mouse anti-human CD19 (HIB19, BioLegend), mouse anti-human CD27 (M-T271, BioLegend), mouse anti-human CD38 (HB-7, BioLegend), mouse anti-human CD24 (ML5, BioLegend), mouse anti-human IgM (MHM-88, BioLegend), mouse anti-human IgD (IA6-2, BioLegend), and mouse anti-human CD138 (MI15, BioLegend). The gating strategy is shown in **Supplementary Figure 1B** for one representative participant. AlexaFluor 647-labeled recombinant human Dsg-3 (extracellular domain, aa 1-566), produced in the baculovirus expression system (31), was included in a separate B-cell staining panel. AlexaFluor 647-labeled recombinant collagen 7, produced in the same way as Dsg-3, was used as a control for a non-specific binding. The gating strategy is shown in **Supplementary Figure 1C** for one representative participant.

### Dsg-Specific B-Cell Detection Using ELISPOT Assay

The frequencies of circulating total IgG and Dsg-specific IgG-antibody secreting cells (ASCs) were determined by human IgG ELISPOT Basic (Mabtech, Sweden) as described (32). In brief, PBMCs from participants in the trial were pre-stimulated with R848 (1 μg/ml) and rhIL2 (10 ng/ml) in complete medium (RPMI-1640 supplemented with 10% FCS, 2 mM L-glutamine, 100 U/ml penicillin, and 100 μg/ml streptomycin [Thermo Fisher Scientific]) in 24-well plates for 72 h at 37°C. Plates of ELISPOT MAIPS-4510 (Merck Millipore, Germany) were coated overnight at 4°C with anti-IgG human antibody. Plates were washed and blocked with complete medium before use. Pre-stimulated PBMCs were washed, resuspended in complete medium, and transferred to the plate, and then incubated for 24 h with 1 × 10^5^ to 4 × 10^5^ PBMCs per well to detect anti-Dsg-1 and -Dsg-3 IgG-secreting ASC, and with 2.5 × 10^3^ to 1 × 10^4^ PBMCs per well to detect total IgG-ASC. IgG-ASCs were detected by addition of biotinylated mouse IgG anti-human IgG. Frequency of anti-Dsg-1 or anti-Dsg-3 IgG ASC was calculated after incubation for 2 h with histidine-tagged recombinant Dsg-1 or Dsg-3 proteins (1 μg/ml) in PBS with Ca^2+^ and Mg^2+^. Biotinylated anti-histidine antibodies (0.5 μg/ml) (Abcam, United Kingdom) were then added. Streptavidin-conjugated peroxidase and tetramethylbenzidine as substrate were used to detect spots. The number of spots were determined using ELISPOT Plate Readers and ImmunoSpot software (CTL Europe GmbH, Germany). Results were expressed as frequencies of Dsg-specific IgG-ASC among total IgG-ASC. The sensitivity and specificity of the ELISPOT assay were estimated at 1 Dsg-3-specific ASC/10^5^ total ASC, and 100%, respectively.

### Statistical Analyses

Descriptive statistical methods were used to analyze data. Summaries (mean, standard error, median, and range) were plotted graphically by study days/time points.

## Results

### Sustained Clinical Response and Autoantibody Suppression

**Figure 1:**
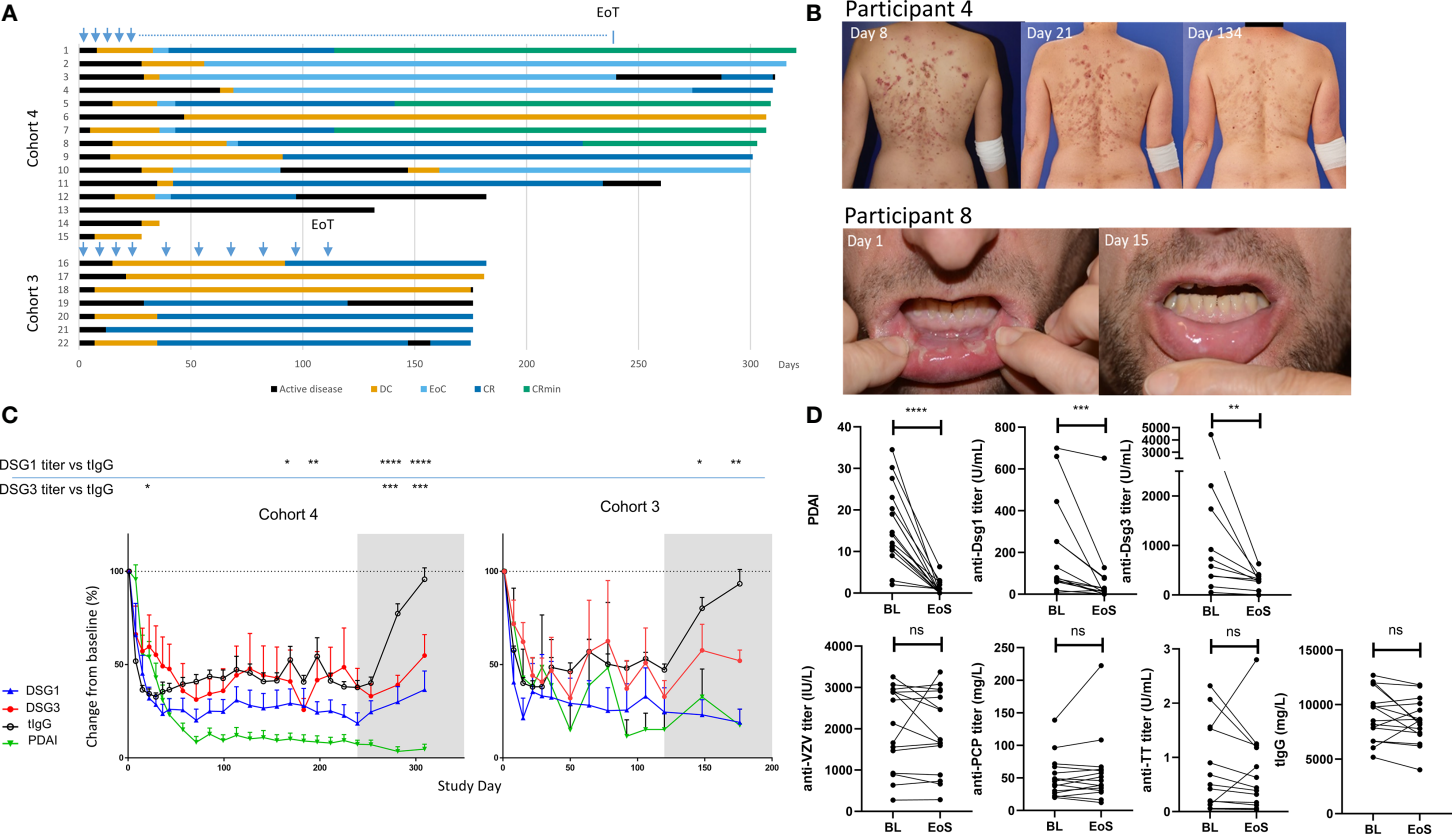
Sustained clinical responses and transient general reduction of total serum IgG levels following efgartigimod treatment. **(A)** Clinical responses to efgartigimod are depicted for all participants in cohorts 3 (*n* = 7) and 4 (*n* = 15). Participant numbers correspond to those used in **Table 1**. Blue arrows indicate when efgartigimod was administered, and the blue dotted line indicates every other week administration time period in participants who achieved EoC. **(B)** Clinical responses of singular representative trial participants with pemphigus foliaceus and pemphigus vulgaris, respectively, are shown. **(C)** Longitudinal changes in pathogenic (anti-Dsg-3 or anti-Dsg-1) as well as total IgG (tIgG) and PDAI activity scores are represented. The gray shaded area indicates efgartigimod treatment-free follow-up period. Means are compared using two-way ANOVA multiple comparison with Dunnett’s post-test. **(D)** Pathogenic (anti-Dsg-3 or anti-Dsg-1), total IgG (tIgG), and non-pathogenic antibodies titers (anti-VZV, anti-varicella zoster virus; TT, tetanus toxoid; PCP, pneumococcal capsular polysaccharide) are compared between BL and EoS for all participants. Wilcoxon matched-pairs test was performed. *p ≤ 0.05; **p ≤ 0.01; ***p ≤ 0.001; ****p ≤ 0.0001. BL, baseline; EoS, end of study; DC, disease control; EoC, end of consolidation; CR, complete clinical remission; CRmin, complete clinical remission with minimal treatment; Pt, participant; ns, non significant.

The characteristics of participants with PV and PF in cohorts 3 and 4, who received extended treatment with efgartigimod (15 and up to 34 weeks, respectively), are shown in **Table 1**. **Figure 1A** shows the clinical outcomes of the patients. Nine participants in cohort 4 (participants 1, 3, 4, 5, 7, 8, 9, 11, and 12) reached CR at any point during the trial while receiving efgartigimod (until day 239) and low-dose prednisone, of which six (participants 1, 4, 5, 7, 8, and 9) were in CR at the end of the study (end of 10-week efgartigimod-free period). Four participants reached complete clinical remission on minimal therapy (CRmin), defined as the absence of new or established lesions while the patient is receiving minimal therapy where minimal therapy is defined as less than or equal to 10 mg/day of prednisone (or the equivalent) for at least 2 months (33), at any point during the study. Participant 3 exhibited low disease activity with a PDAI activity score of 3 following CR and two participants (11 and 12) relapsed to a PDAI activity score >10. Three participants (2, 6, and 10) improved clinically and maintained clinical improvement status until end of study: participants 2 and 10 reached and maintained EoC, and participant 6 reached DC and had a PDAI activity score of 1 at end of the study. In cohort 3, five of seven participants achieved CR at any point during the study (participants 16, 19, 20, 21, and 22), and four of these participants were in CR at the end of treatment-free follow-up (participants 16, 20, 21, and 22).

**Table 1:** Baseline characteristics of participants receiving extended efgartigimod treatment in cohorts 3 and 4.

Participant		Pemphigus Disease History	Gender	Age	Type of Pemphigus	PDAI Activity at Baseline	Subanalyses
Cohort 4							
**1** 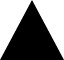		Relapsing	F	63	PF	9	S, CI
**2** 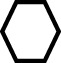		Relapsing	F	48	PV M	27.6	S
**3** 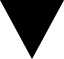		Relapsing	M	57	PF	20.3	S, CI
**4** 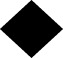		Newly diagnosed	F	42	PF	34.5	S, CI, P
**5** 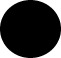		Relapsing	F	62	PV M	14.6	S, CI
**6** 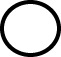		Relapsing	M	66	PV MC	11	S, CI
**7** 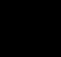		Relapsing	F	85	PF	11.2	S, CI
**8** 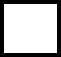		Newly diagnosed	M	47	PV MC	10.3	S, CI, P
**9** 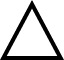		Newly diagnosed	M	67	PF	19	S, CI
**10** 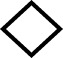		Relapsing	F	36	PF	30.2	S, CI
**11** 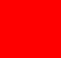		Newly diagnosed	M	58	PV C	2	CI
**12** 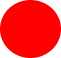		Newly diagnosed	F	66	PV MC	7.3	CI
**13** 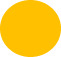		Newly diagnosed	F	51	PF	30.3	CI
**14**		Newly diagnosed	M	22	PV MC	39.9	
**15**		Relapsing	M	30	PV C	28.4	
Cohort 3							
**16**		Newly diagnosed	F	40	PV M	2	
**17**		Relapsing	M	36	PV MC	3	
**18**		Relapsing	F	48	PV M	12	
**19**		Relapsing	F	52	PV M	1	
**20** 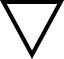		Newly diagnosed	F	65	PV MC	23	S
**21** 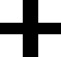		Relapsing	F	30	PV MC	18.9	S
**22**		Relapsing	F	54	PV MC	14	

PDAI, pemphigus disease area index; PV, pemphigus vulgaris; PF, pemphigus foliaceus; M, mucosal-dominant; MC, mucocutaneous; C, cutaneous.

The subanalyses column indicates the type of analysis performed: S, serology; CI, cellular immunity; P, photography. List of participants, pemphigus disease history, gender, age, type of pemphigus, and PDAI activity at baseline. The subanalyses column indicate the type of analysis performed: S, serology; CI, cellular immunity; P, photography. Figure symbol shapes used throughout the manuscript are indicated for each participant. PDAI, pemphigus disease area index; PV, pemphigus vulgaris; PF, pemphigus foliaceus; M, mucosal-dominant; MC, mucocutaneous; C, cutaneous.

In order to characterize the observed sustained clinical responses, representative participants with PV (participant 8) and PF (participant 4) from cohort 4 are shown in **Figure 1B**. Participant 8 with PV who had a PDAI activity score of 10.3 at baseline showed a decline of disease activity starting after the first treatment and mucosal blisters had healed by day 15. Skin lesions of participant 4 with PF who had baseline PDAI activity of 34.5 declined gradually over time and resolved completely by day 281. Importantly, both participants showed no disease activity during the 10-week observation period after last efgartigimod treatment.

In the participants with sustained clinical response (whose PDAI activity and serological responses are summarized in **Supplementary Table 1**), total serum IgG and pathogenic autoantibodies declined early. Total IgG levels remained suppressed to about 70% during the treatment period, and recovered to baseline levels during treatment-free follow-up, in line with what has been previously reported for the overall study population (28). Anti-Dsg antibodies declined early and prolonged reductions were observed in all responding participants (**Figure 1C**). Unlike protective antibody titers (anti-VZV, anti-TT, and anti-PCP) that returned to baseline levels at the end of the study, stable suppression of anti-Dsg-1 antibodies and anti-Dsg-3 antibodies to lower levels than before treatment were observed during the 10 weeks of efgartigimod-free follow-up (**Figure 1D**).

### Suppression of Anti-Dsg Autoantibody Subtypes

Since PV and PF primarily involve IgG4 (34), and other IgG subtypes to a lesser extent, ALBIA Dsg-3 assay was performed in six participants with PV, and modified anti-Dsg-1 Euroimmun ELISA in six participants with PF who received extended efgartigimod treatment in cohorts 3 and 4 and achieved a sustained clinical response (28). Participants evaluated in these analyses are specified in **Table 1**. At baseline, we detected heterogenous anti-Dsg-1/3 IgG1-4 subclasses in different participants. In participants with baseline levels above the positivity threshold, we observed reduction of anti-Dsg-3 IgG1 autoantibodies to below the positivity threshold in 3 of 3 participants and anti-Dsg-3 IgG4 in 2 of 3 participants (**Supplementary Figure 2A**). Anti-Dsg-3 IgG2 autoantibodies, present in 3 participants, were markedly reduced although only 1 subject decreased below the positivity threshold at end of the study; IgG3 autoantibodies present in one participant at baseline became suppressed below the positivity threshold. The six participants with PF subjected to subclass analysis demonstrated a dominant IgG4 signature of anti-Dsg-1 autoantibodies (**Supplementary Figure 2B**), while one participant presented with IgG1 autoantibodies. In this patient population, analysis of Dsg-1 IgG titers over time showed reduction of Dsg-1-specific IgG1 and IgG4 antibodies following clinical response, which remained suppressed at the end of the study. Thus, decreases in anti-Dsg titer regardless of the IgG subtype analyzed were observed following efgartigimod treatment in this subpopulation.

### Suppression of Circulating Immune Complexes

Immunocomplexes are found in healthy individuals (35), but their formation can be expected to be elevated in autoimmune diseases and partly drive and/or exaggerate their pathologies (36). As the half-life of immune complexes, largely containing IgG, may also be affected by the biology of FcRn (35), we investigated levels of CIC in the same six participants with PV and six participants with PF. These participants were subjected to IgG CIC analysis by C1q ELISA, which detects complement-fixed IgG antibodies (without providing information about antigen specificity of CICs). IgG CIC levels are considered clinically significant if ≥4.0 μg Eq/ml. Four participants presented with elevated CIC levels, but notable reduction of CICs during treatment was observed (**Supplementary Figures 3A, B**), consistent with the observed improvement in their clinical condition. Rise of CICs following efgartigimod discontinuation did not lead to rise in disease activity, suggesting that for these participants, CICs were not directly linked with the disease activity.

### Changes in Dsg-3+ and Dsg-1+ B Cells

The observed differences of anti-Dsg-1/3 titers compared to overall serum IgG prompted us to probe the PBMCs of participants with PV to determine the fate of Dsg-3-specific B cells that could potentially explain the prolonged suppression of anti-Dsg autoantibodies. PBMCs were collected in cohort 4 participants only (*n* = 15), and were successfully isolated in 12 participants (5 PV, 7 PF), of whom nine achieved a sustained clinical response (3 PV, 6 PF).

**Figure 2:**
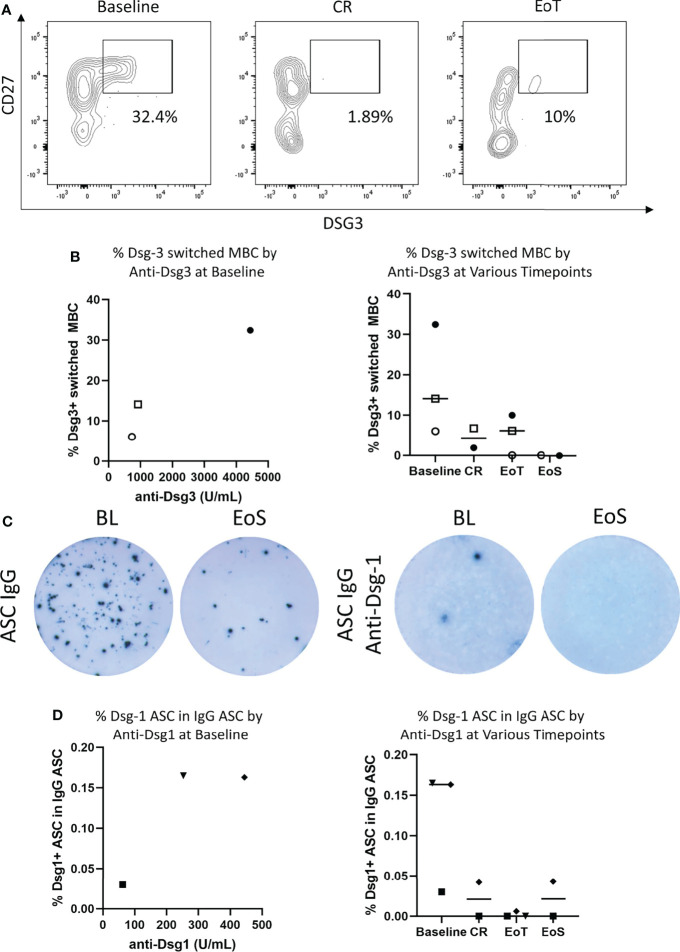
Efgartigimod reduces antigen-specific B cells in the blood. **(A)** Among the three PV participants with viable PBMC and sustained clinical response, representative flow cytometry plots depicting frequency of Dsg-3+ switched memory B cells (MBC) for one participant with PV at baseline, CR, and EoT were generated. **(B)** Frequency of circulating Dsg-3+ switched memory B cells in three participants with PV with sustained clinical response during the study in relation to Dsg-3 autoantibody serum titers at baseline and by time point. **(C)** Among the three PF participants with viable PBMCs, a representative ELISPOT of total IgG-ASCs with 2.5 × 10^3^ to 1 × 10^4^ PBMCs plated per well (left) and Dsg-1 IgG ASCs with 1 × 10^5^ to 4 × 10^5^ PBMCs plated per well (right) detected in PBMCs of a participant with PF. **(D)** Frequency of peripheral blood Dsg-1-specific ASCs evaluated by ELISPOT assay in 3 participants with PF during study both in relation to Dsg-1 autoantibody serum titers at baseline and by time point. Frequencies are reported as a percentage of total IgG ASC. ASCs, antibody-secreting cells; BL, baseline; CR, complete clinical remission; EoT, end of treatment; EoS, end of study.

Frequency of Dsg-3+ B cells in the periphery was identified by staining for CD45+, CD19+, and CD27+ memory B cells (MBCs) and fluorescently labeled Dsg-3 antigen (**Figure 2A**) as previously described (13). Additional staining with IgM and IgD was included prior to gating on Dsg-3+ cells to identify class-switched memory B cells. Dsg-3+ B cells were identified within CD27+ IgM- IgD- cells, known to harbor antigen-specific B cells (37, 38) (**Figure 2B**). In line with previous reports, Dsg-3-specific B cells were rarely seen in peripheral blood but were detected principally at baseline and were detected at higher frequencies in participants with higher anti-Dsg-3 serum IgG levels in the samples analyzed. A reduction of antigen-specific MBCs was visible when participants 5, 6, and 8 reached CR and at the end of treatment. Two PV participants that relapsed following CR also exhibited reduction of Ag-specific B cells at CR (**Supplementary Figure 4**) and no increase in frequency of Dsg-3+ B cells was further observed, possibly implicating persistent tissue-resident antigen-specific responses in relapse (39).

In addition to assessing the frequency of Dsg-3+ B cells in participants with PV, Dsg-1 specific ASCs were measured by ELISPOT. Although ELISPOT assays were performed in all samples, low cell viability after thawing and withdrawal of participant’s consent for post-hoc analysis restricted data analysis to three participants who are presented here (participants 3, 4, and 7). At baseline, anti-Dsg-1 IgG ASCs were detected in all 3 PF participants assessed and were detected at higher frequency in participants with higher serum anti-Dsg1 levels (**Figures 2C, D**). Following treatment with efgartigimod and concomitant low-dose prednisone, anti-Dsg-1 IgG ASCs were no longer detectable at the end of the study. For two participants, disappearance of anti-Dsg-1 IgG ASC was detected at the CR visit (on days 44 and 275, respectively). One participant demonstrated loss of anti-Dsg-1 IgG ASC at the end of treatment, while CR was achieved 47 days later during treatment-free follow-up.

### T-Cell and B-Cell Phenotypes During Efgartigimod Treatment

**Figure 3:**
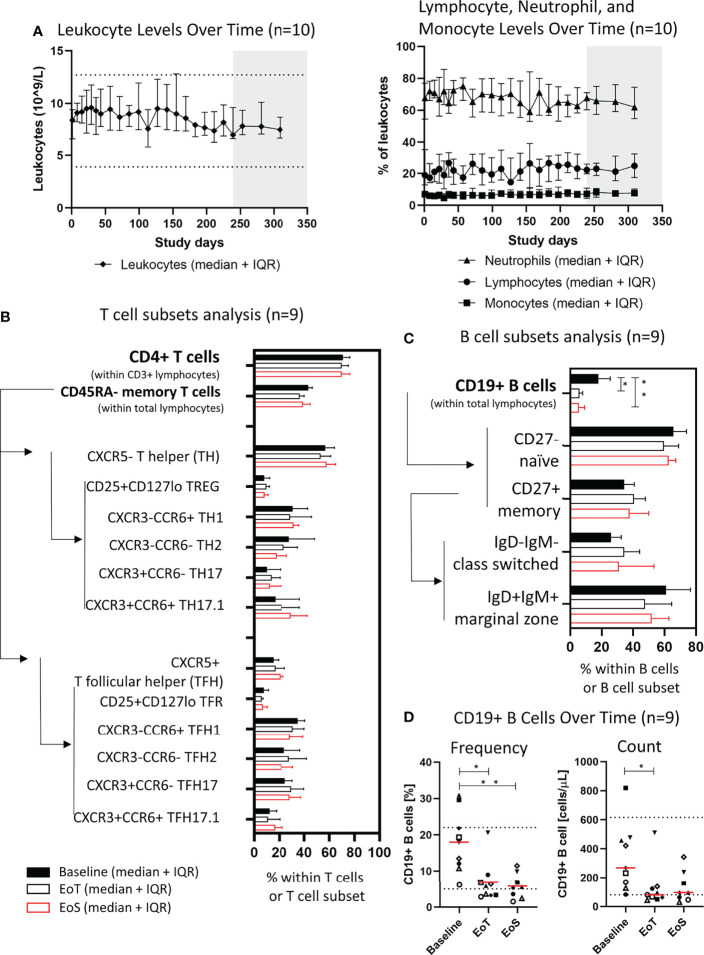
Efgartigimod does not affect frequencies of lymphocyte subsets except B cells. **(A)** Leukocytes (10^9^/L) and frequency (%) of neutrophils, lymphocytes, and monocytes within leukocytes in peripheral blood of cohort 4 participants (*n* = 10), median, and IQR are plotted. **(B)** Frequency of CD4+ T cells within lymphocytes and their subsets of cohort 4 participants with viable PBMC (*n* = 9), median, and IQR are plotted. **(C)** Frequency of CD19+ B cells within lymphocytes and their subsets of cohort 4 participants with viable PBMC (*n* = 9), median, and IQR are plotted. Arrows and lines indicate the way the frequency of a parent population was determined. **(D)** Frequency and counts of CD19+ B cells at baseline, EoT, and EoS of cohort 4 participants with viable PBMC and sustained clinical response (*n* = 9). Red lines represent medians and dotted lines represent normal limits. A non-parametric one-way ANOVA with Dunn’s post-test was performed. *p ≤ 0.05; **p ≤ 0.01. EoT, end of treatment; EoS, end of study; IQR, interquartile range.

Beyond autoantibody-producing B cells, classical type-2 T helper (Th2) cells are typically considered central players in pemphigus pathology; however, recent advances in clinical immunology pointed to additional T-cell subsets, namely, T helper-17/T follicular helper-17 (Th17/Tfh17) cells efficiently promoting autoantibody production in participants with pemphigus (7, 8, 40). Thus, we then investigated whether prolonged treatment had an impact on major T-cell and B-cell subsets in addition to the antigen-specific context. Importantly, treatment did not impact global levels of total leukocytes, total lymphocytes, monocytes, or neutrophils (**Figure 3A**). Longitudinal analysis of PBMCs at 4 different time points in the 9 participants from cohort 4 with viable PBMC did not reveal changes in median overall T-cell subpopulations. CD4+ T cells and subsets were stable with no significant changes in Th1, Th2, Th17 or Tfh1, Tfh2, Tfh17 subsets observed (**Figure 3B**).

Baseline frequency of total B cells as a proportion of total lymphocytes was heterogenous and ranged from 6.2% to 30.9%, indicating high B-cell frequency in some individuals possibly due to B-cell activation and proliferation. Analysis of B cells revealed declining numbers of median total CD19+ B cells in the periphery of all nine participants but without affecting the composition of B-cell subsets for the markers tested including CD27+ memory cells (**Figures 3C, D**). After treatment, median levels of CD19+ B cells remained within normal ranges.

The highest B-cell frequencies in the trial were observed in participants 11 and 12 who relapsed to PDAI activity >10 after having achieved CR. At the last evaluable time point, no increases from baseline in B-cell frequency were noted. Participant 13 with PF showed no clinical response to efgartigimod and did not demonstrate changes in CD19+ B cells (**Supplementary Figure 5**).

### Prednisone Exposure in Participants With Prolonged Efgartigimod Treatment

**Figure 4:**
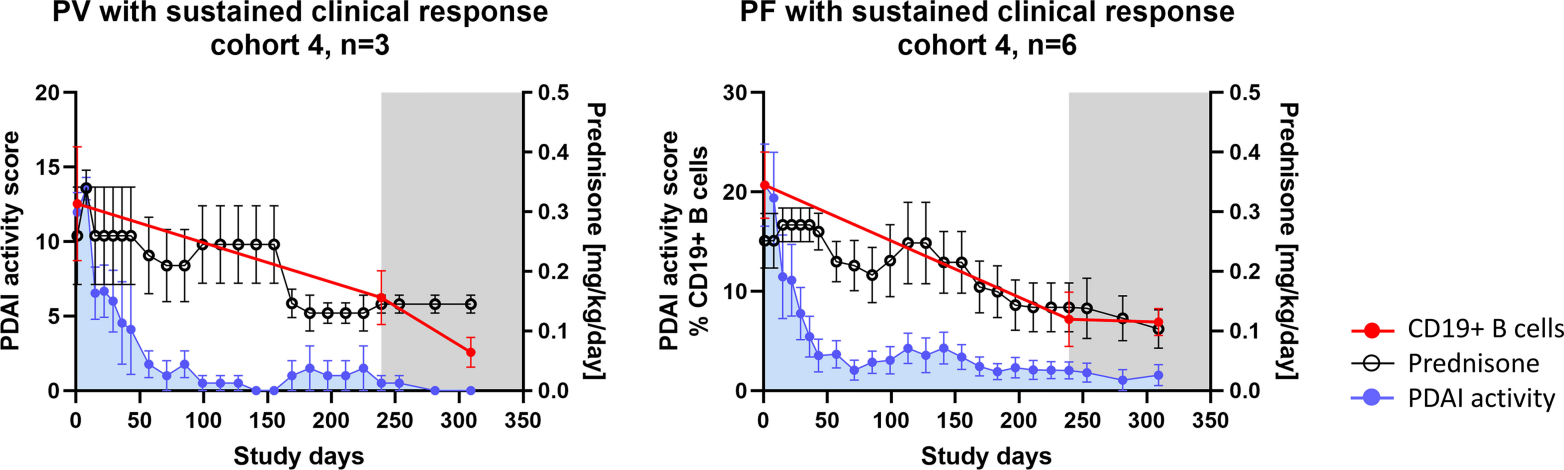
Prednisone exposure in participants with prolonged efgartigimod treatment and observed B-cell immunomodulation (*n* = 9). Summary profiles of the PV and PF participants achieving sustained clinical response illustrating prednisone dosages, PDAI activity scores, and frequency of total CD19+ B cells in peripheral blood when available. The gray shaded area indicates efgartigimod-free follow-up period. Error bars represent standard error of the mean (SEM). PV, pemphigus vulgaris; PF, pemphigus foliaceus; PDAI, pemphigus disease area index.

In order to investigate the potential impact of prednisone on the observed pharmacodynamic and immunological effects described, concomitant prednisone exposure was analyzed. As seen in the overall study population, early clinical efficacy of efgartigimod allowed for early tapering of steroids. This effect was further pronounced in participants who achieved sustained clinical response after prolonged treatment with efgartigimod in cohorts 3 and 4. In participants who achieved CR at any point in the study, the average daily prednisone dose until CR was 0.255 mg/kg/day for cohort 4 participants (*n* = 9) and 0.18 mg/kg/day for cohort 3 participants (*n* = 5) (**Table 2**), and reduced frequencies of B cells were not associated with higher prednisone dosages (**Figure 4**).

**Table 2:** Clinical status and concomitant prednisone dose levels over time in cohorts 3 and 4.

Type of pemphigus		PDAI activity at baseline	Delay to first relapse after DC (days)	Dose of prednisone at the time of first relapse (mg/kg/day)	Clinical status at the last evaluation/PDAI activity	Initial dose prednisone (mg/kg/day)	Dose of prednisone at the last evaluation (mg/kg/day)	Average daily prednisone dose until CR (mg/kg/day)	Average daily prednisone dose over the study (mg/kg/day)
Cohort 4									
**1**	PF	9			CR/0	0.28	0	0.273	0.080
**2**	PV M	27.6			EoC/2	0.28	0.35		0.278
**3**	PF	20.3	211	0.08	AD/3	0.24	0.06	0.111	0.108
**4**	PF	34.5			CR/0	0.31	0.11	0.218	0.207
**5**	PV M	14.6			CR/0	0.37	0.13	0.370	0.194
**6**	PV MC	11			DC/1	0.10	0.10		0.100
**7**	PF	11.2			CR/0	0.22	0.06	0.215	0.100
**8**	PV MC	10.3			CR/0	0.31	0.16	0.310	0.243
**9**	PF	19			CR/0	0.06	0.19	0.235	0.223
**10**	PF	30.2	63	0.20	EoC/6.3	0.40	0.20		0.318
**11**	PV C	2	200	0.23	AD/27.9	0.23	1.8	0.230	0.347
**12**	PV MC	7.3	82	0.08	AD/10.9	0.33	0.98	0.330	0.310
**13**	PF	30.3			-/17	0.32	0.32		0.320
**14**	PV MC	39.9			DC/23.2	0.33	1.3		0.943
**15**	PV C	28.4			DC/17.8	0.61	0.61		0.610
Cohort 3									
**16**	PV M	2			DC/1	0	0.06	0.027	0.071
**17**	PV MC	3			DC/1	0.14	0.14		0.134
**18**	PV M	12	169	0.09	AD/2.6	0.54	0.09		0.123
**19**	PV M	1	92	0.17	AD/3.3	0.06	0.65	0.060	0.188
**20**	PV MC	23			CR/0	0	0.27	0.053	0.226
**21**	PV MC	18.9			CR/0	0.48	0.48	0.480	0.480
**22**	PV MC	14	141	0.07	CR/0	0.28	0.14	0.280	0.140

PV, pemphigus vulgaris; PF, pemphigus foliaceus; M, mucosal-dominant; MC, mucocutaneous; C, cutaneous; DC, disease control; EoC, end of consolidation; CR, complete clinical remission; AD, active disease.

## Discussion

Reduction of IgG levels by efgartigimod resulted in early and durable clinical responses in participants with pemphigus (28). In this secondary analysis of the open-label phase 2 study, we have found for the first time a possible immunomodulatory effect by an anti-FcRn inhibitor beyond blocking of IgG recycling, as evidenced by the observed prolonged reduction of autoantibody levels during the 10 weeks of efgartigimod-free follow-up and indications of disease modification in peripheral lymphocytes in some patients. In addition, the early clinical improvement of participants (28) allowed for early prednisone tapering, which led to a comparatively lower corticosteroid exposure throughout the study than in the general pemphigus population (21).

Clinical benefit in participants was directly linked to decreasing titers of pathogenic autoantibodies, as previously described (28, 41). Reduction of anti-Dsg-1 IgG was more consistent across participants, while changes in anti-Dsg-3 IgG levels were more variable. Of note, it has been shown earlier that anti-Dsg-3 levels do not always correlate with disease activity (42, 43). Persistent re-augmentation or high levels of anti-Dsg-1 antibodies correlate closely with the development of skin relapses, whereas anti-Dsg-3 antibodies are less specific, and may occasionally be observed in some PV patients in clinical remission (44). The pathogenic effects of anti-Dsg antibodies have predominantly been attributed to Fab-mediated function (45) causing steric hindrance, depletion of Dsgs, and acantholysis (21). Moreover, involvement of Fc-mediated complement activation has also been described (46), and elevated levels of circulating immune complexes are considered to contribute to more severe disease (47). The predominance of IgG4 subclass anti-Dsg antibodies (34) implies limited engagement of the effector functions, yet IgG4 antibodies can activate *via* FcγRI and FcγRIIa (48–50) expressed by skin macrophages (51). Interestingly, anti-Dsg-1/3 IgG1 and other subclass responses were found at baseline in several participants, indicating that these may be more common than expected from previous reports given the small number of participants analyzed, and confirming that certain heterogeneity of autoimmune profiles exists. Together, these data suggest that both FcγR- and complement-mediated effector functions of anti-Dsg IgG antibodies may also play a role in pemphigus pathology while removal of all IgGs regardless of their subclasses by anti-FcRn therapy has the potential to limit the effect of all pathogenic antibody functions, both Fab- and Fc-mediated, which may also translate into clinical benefits as demonstrated by these results.

Pathogenic anti-Dsg antibodies are thought to originate from continuously generated short-lived plasma cells (SLPCs) that require a persistent inflammatory milieu to exist (14, 52). Moreover, Dsg-positive B cells show a more activated phenotype than their Dsg-negative counterparts (53). We observed that Dsg-1/3 antigen-specific B cells were decreased following efgartigimod treatment, suggesting that this therapy can trigger an immunomodulatory effect by reintroducing a long-term immune homeostasis beyond the direct effect of reducing autoantibody levels. As analysis of B-cell subsets revealed no changes, the modulation of total CD19+ B cells observed may be driven by this decrease in antigen-specific B cells, both by Dsg-autoreactive B cells measured in our study and by other antigen-specific B cells not measured here, or may result from a change in a specific B-cell subpopulation that was not studied here. It remains to be investigated by which mechanism(s) efgartigimod treatment is impacting antigen-specific B-cell signatures and whether this effect will be observed in other diseases treated with efgartigimod. Whether removal of autoantibodies exerts an indirect effect on autoreactive B cells that no longer receive immune stimulation or a direct effect, especially since peripheral blood (54) and splenic B cells express FcRn (55), remains to be elucidated. Alternatively, efgartigimod may also modulate antigen presentation by other cells such as dendritic cells (18), or may impact still unexplored immune pathways. The contribution of prednisone to the immunomodulatory effect on the B-cell compartment remains to be investigated in a placebo-controlled study, as impact of corticosteroid treatment is broad and can induce variable responses also impacting B-cell autoimmune signature (56). Since PV lesions contain Dsg-specific B cells and increased concentrations of CD19+ B cells (39), studies are needed to evaluate potential effects of efgartigimod in tissues.

The activation of B cells results in their proliferation and eventually differentiation into ASCs and MBCs. A single B cell may, within a week, give rise to as many as 5,000 ASCs. Expansion of specific B-cell subpopulations has been reported in autoimmunity (57–59), including pemphigus (60, 61), where CD19-high B cells are found to be elevated. While we could not distinguish the CD19-high subset (data not shown), baseline levels of total CD19+ B cells were enriched in some study participants. Whether these constituted expansions of antigen-specific B cells or other particular B-cell subsets is currently unknown. Nevertheless, a decrease of CD19+ B cells frequency towards a return to normality (approximately 10% of lymphocytes) may become a promising tool to explore as a proxy of regained immune homeostasis and indicate time to discontinue efgartigimod therapy.

Targeted immune disengagement, whether at the level of autoantibodies or upstream at the level of autoantibody-producing B cells, appears to be a sensible approach to tackle IgG-mediated autoimmunity and offers an opportunity to decrease usage of broad immunosuppression, including corticosteroids. B-cell depleting antibody treatments targeting CD20 [such as rituximab (4, 5)] are used and explored as therapeutic options. However, because rituximab has a relatively slow onset of action in reducing autoantibody levels, achievement of disease control, an early clinical endpoint, relies on the use of corticosteroids. Other approaches that interfere with B-cell signaling, e.g., acting on Bruton’s tyrosine kinase (BTK) signaling, are also being explored as therapeutic approaches in PV. A phase 2 study using the BTK-inhibitor rilzabrutinib demonstrated steroid reduction to a minimal dose (≤10 mg per day) after approximately 16 weeks with 22% of participants reaching CR by week 24 (62), yet rilzabrutinib failed to meet its primary or key secondary endpoints in a phase 3 trial (63). In the phase 2 study with efgartigimod, 64% of participants treated with efgartigimod reached CR with a prednisone dose less than 0.5 mg/kg/day in a median time of 13 weeks. Nine of 15 participants in cohort 4 achieved CR at any point during the study and 6 were still in CR at the end of treatment-free follow-up. Notably, the 4 patients who achieved CRmin maintained this condition until the end of the study. In cohort 3, 5 of the seven participants achieved CR at any point during the study and 4 of these participants were in CR at the end of treatment-free follow-up. Following this phase 2 study, a phase 3 randomized controlled trial was initiated to further study the efficacy and safety of efgartigimod in PV and PF (NCT04598451).

Given the complexity of pemphigus pathology with the involvement of innate immunity, T cells, B cells, and autoantibodies, it is not surprising that various therapies beyond those targeting autoantibody-producing B cells are also being investigated [reviewed in (20)]. It remains to be determined how potent these approaches are, individually and collectively, and whether complete elimination of corticosteroids and broad immunosuppressants can be achieved. The ongoing phase 3 trial with efgartigimod in PV and PF will determine how FcRn antagonism fits in the treatment paradigm of pemphigus.

Limitations of this secondary analysis include those associated with open label studies, lack of placebo control, small sample size, and post-hoc analyses. PBMC collection was implemented in cohort 4 participants only following clinical observations from earlier cohorts and expert recommendations to expand understanding of efgartigimod’s impact on cellular and serological immunity. For few patients, PBMC samples with sufficient viability were available, allowing for longitudinal analyses. Future studies with larger sample sizes and placebo control are underway, which will further explore these preliminary observations.

In conclusion, our data demonstrate differential impact of efgartigimod on protective and autoreactive anti-Dsg antibodies with more potent inhibition of the latter. Moreover, we demonstrate that the therapeutic effect of FcRn inhibition by efgartigimod may extend beyond blocking of IgG recycling and appears to also exert effects on the B-cell compartment. This combined with the early onset of clinical efficacy places efgartigimod in a unique position to potentially target multiple IgG-mediated immune disorders in a well-tolerated, precise, and potentially disease-modifying manner. Further studies in larger populations with a placebo comparison are needed to elucidate this observed phenomenon.

## Data Availability Statement

Argenx is committed to responsible data sharing regarding the clinical trials they fund. Included in this commitment is access to anonymized, individual, and trial-level data (analysis datasets), and other information (e.g., protocols and clinical study reports), as long as the trials are not part of an ongoing or planned regulatory submission. These clinical trial data can be requested by qualified researchers who engage in rigorous independent scientific research and will only be provided after review and approval of a research proposal and statistical analysis plan and execution of a data sharing agreement. Data requests can be submitted at any time, and the data will be accessible for 12 months. Requests can be submitted to ESR@argenx.com.

## Ethics Statement

The studies involving human participants were reviewed and approved by relevant ethics committees of each participating center (28). The patients/participants provided their written informed consent to participate in this study. Written informed consent was obtained from the individual(s) for the publication of any potentially identifiable images or data included in this article.

## Author Contribution

MM-V, M-LG, MS, JS, PV, MG, ZB-C, PJ, and SC contributed to the design of the study. MM-V, M-LG, SC, and MH performed the experiments. MM-V, M-LG, SC, MS, BB, and PV analyzed data. MM-V, M-LG, SC, PJ, GV, MS, BB, and PV have discussed and interpreted the data. MS wrote the first draft of the manuscript. PV, BB, SC, and GV wrote sections of the manuscript. All authors critically revised the manuscript, read, and approved the submitted version.

## Conflict of Interest

ZB-C reports consulting fees for Sanofi-Genzyme, Novartis, and argenx. MG has served as a consultant and on advisory boards for argenx, Biotest, GSK, Janssen, LEO Pharma, Lilly, Novartis, and UCB. MS, BB, and PV are employed by argenx. PJ has served as a consultant for Amgen, Principia Biopharma, argenx, AstraZeneca, Janssen, Thermo Fisher, Lilly, Sanofi, Akari, Chugai, Novartis, Kezar, Genentech, and Topas. GV is a paid consultant for argenx. SC, MM-V, and M-LG as employees of INSERM U1234, Normandie University have a collaborative research agreement with argenx.

The remaining authors declare that the research was conducted in the absence of any commercial or financial relationships that could be construed as a potential conflict of interest.

This study received funding from Argenx. The funder had the following involvement with the study: participated in the study design, research, analysis, data collection, interpretation of data, and review and approval of the publication. Argenx also funded the medical writing support of this manuscript.

## Publisher’s Note

All claims expressed in this article are solely those of the authors and do not necessarily represent those of their affiliated organizations, or those of the publisher, the editors and the reviewers. Any product that may be evaluated in this article, or claim that may be made by its manufacturer, is not guaranteed or endorsed by the publisher.

## Supplementary Material

Supplementary Figure 1
**(A)** Gating strategy to study CD4+ helper T cells and their subsets. **(B)** Gating strategy to study CD19+ B cells and their subsets. **(C)** Gating strategy to study CD19+ IgM- IgD- CD27+ DSG3+ or Col7+ cells for one representative participant. Col7 was used as a control for a non-specific binding.

Supplementary Figure 2 Efgartigimod results in reduction of autoantibodies of all subclasses. **(A)** Dsg-3-specific IgG1, IgG2, IgG3 and IgG4 serum titers at baseline, CR, EoT, and EoS in participants with PV (n = 6). **(B)** Dsg-1-specific IgG1 and IgG4 serum titers at baseline, CR, EoT, and EoS in participants with PF (n = 6) Black lines indicate median. Dotted line indicates the threshold of positivity (2 SD above mean value from 36 healthy donors for **(A)** 20 RU/mL for **(B)** CR, complete clinical remission; EoT, end of treatment; EoS, end of study.

Supplementary Figure 3 Efgartigimod transiently reduces levels of circulating immune complexes. Levels of IgG circulating immune complexes (IgG CIC) levels in participants with PV (n = 6) **(A)** and PF (n = 6) **(B)** following efgartigimod treatment. Black lines indicate median. Dotted line indicates CS (clinical significance). CIC, circulating immune complexes; CR, complete clinical remission; EoT, end of treatment; EoS, end of study; PV, pemphigus vulgaris; PF, pemphigus foliaceus. Time points for CR are variable based on individual participant response, and are indicated in **Figure 1**.

Supplementary Figure 4
**(A)** Antibody titer of anti-Dsg-3 antibodies and **(B)** frequency of circulating Dsg-3+ switched memory B cells (MBC) in the five participants with PV (3 with sustained clinical response shown in **Figure 3B**, and 2 with relapse following CR). Black lines indicate median. CR, complete clinical remission; EoT, end of treatment.

Supplementary Figure 5 Frequency of CD19+ B cells in PV and PF participants with sustained clinical response, those relapsing following CR, and one with no clinical response during the study. Red or black lines indicate median. Dotted lines represent normal limits. CR, complete clinical remission; EoT, end of treatment.
