# Supplementary material for: FcRn Antagonism Leads to a Decrease of Desmoglein-Specific B Cells: Secondary Analysis of a Phase 2 Study of Efgartigimod in Pemphigus Vulgaris and Pemphigus Foliaceus
Source: Front Immunol. 2022 May 18;13:863095. doi: 10.3389/fimmu.2022.863095 (PMC9157593; doi:10.3389/fimmu.2022.863095)
Supplement: Supplementary Figure 1 — (A) Gating strategy to study CD4+ helper T cells and their subsets. (B) Gating strategy to study CD19+ B cells and their subsets. (C) Gating strategy to study CD19+ IgM- IgD- CD27+ DSG3+ or Col7+ cells for one representative participant. Col7 was used as a control for a non-specific binding. [file Presentation_1.pdf]

Supplemental Figure 1

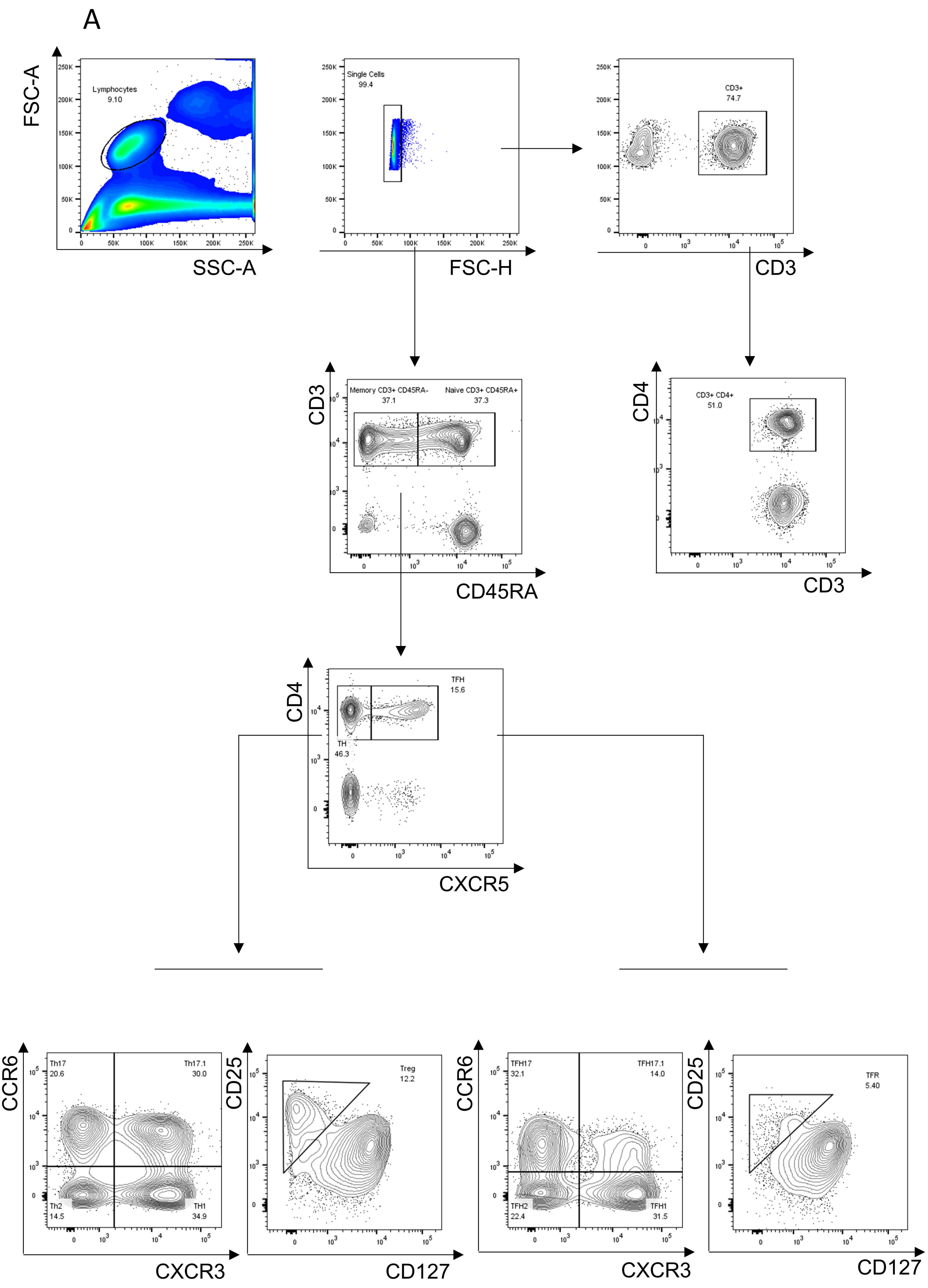

B

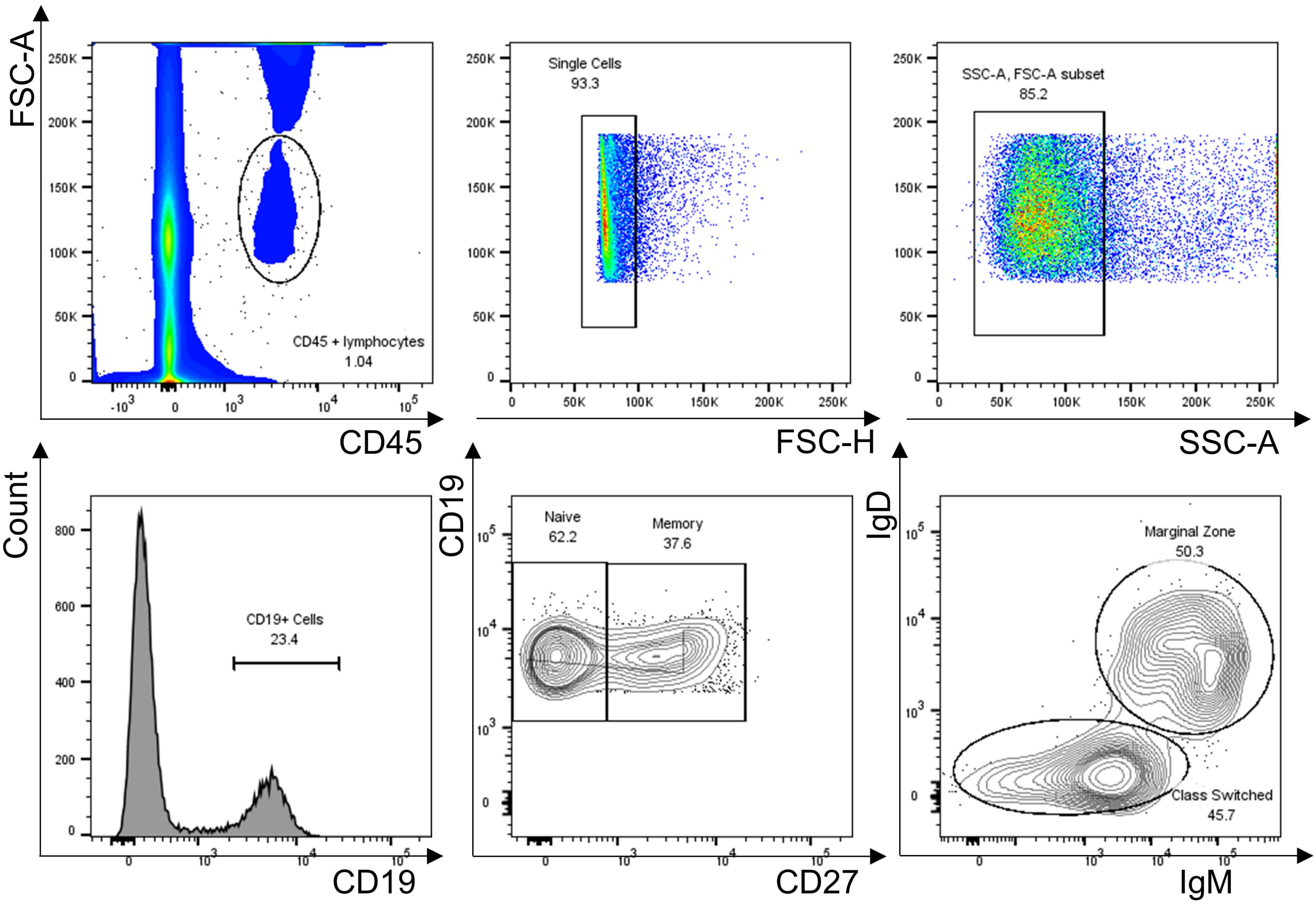
FSC-A
CD45 + lymphocytes
1.04
CD45
Single Cells
93.3
FSC-H
SSC-A, FSC-A subset
85.2
SSC-A
Count
CD19+ Cells
23.4
CD19
CD19
Naive
62.2
Memory
37.6
CD27
IgD
Marginal Zone
50.3
Class Switched
45.7
IgM

C

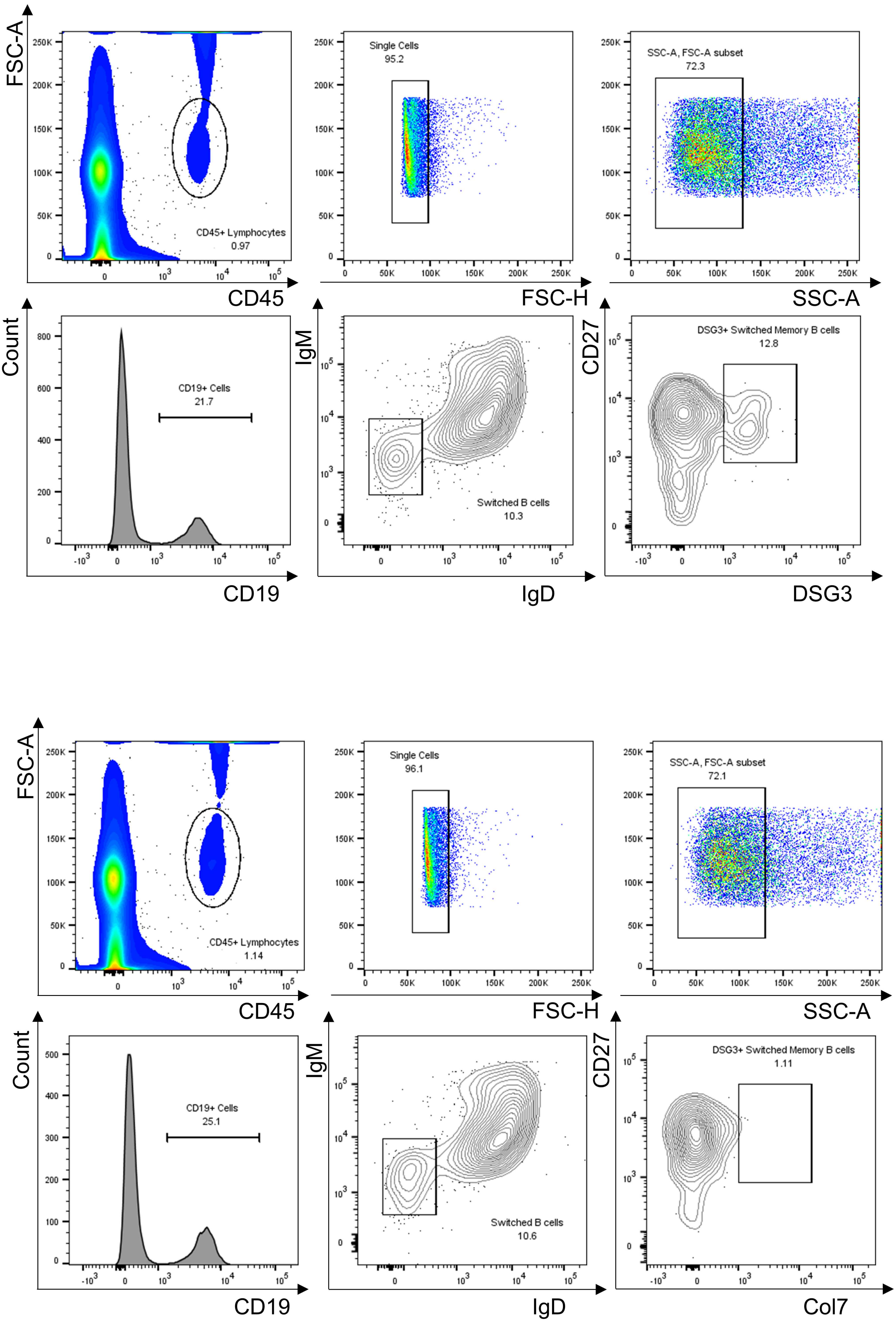

FSC-A
CD45
CD45+ Lymphocytes
0.97
Single Cells
95.2
FSC-H
SSC-A, FSC-A subset
72.3
SSC-A
Count
CD19+ Cells
21.7
CD19
IgM
Switched B cells
10.3
IgD
CD27
DSG3+ Switched Memory B cells
12.8
DSG3
FSC-A
CD45
CD45+ Lymphocytes
1.14
Single Cells
96.1
FSC-H
SSC-A, FSC-A subset
72.1
SSC-A
Count
CD19+ Cells
25.1
CD19
IgM
Switched B cells
10.6
IgD
CD27
DSG3+ Switched Memory B cells
1.11
Col7

Supplemental Figure 2.

A

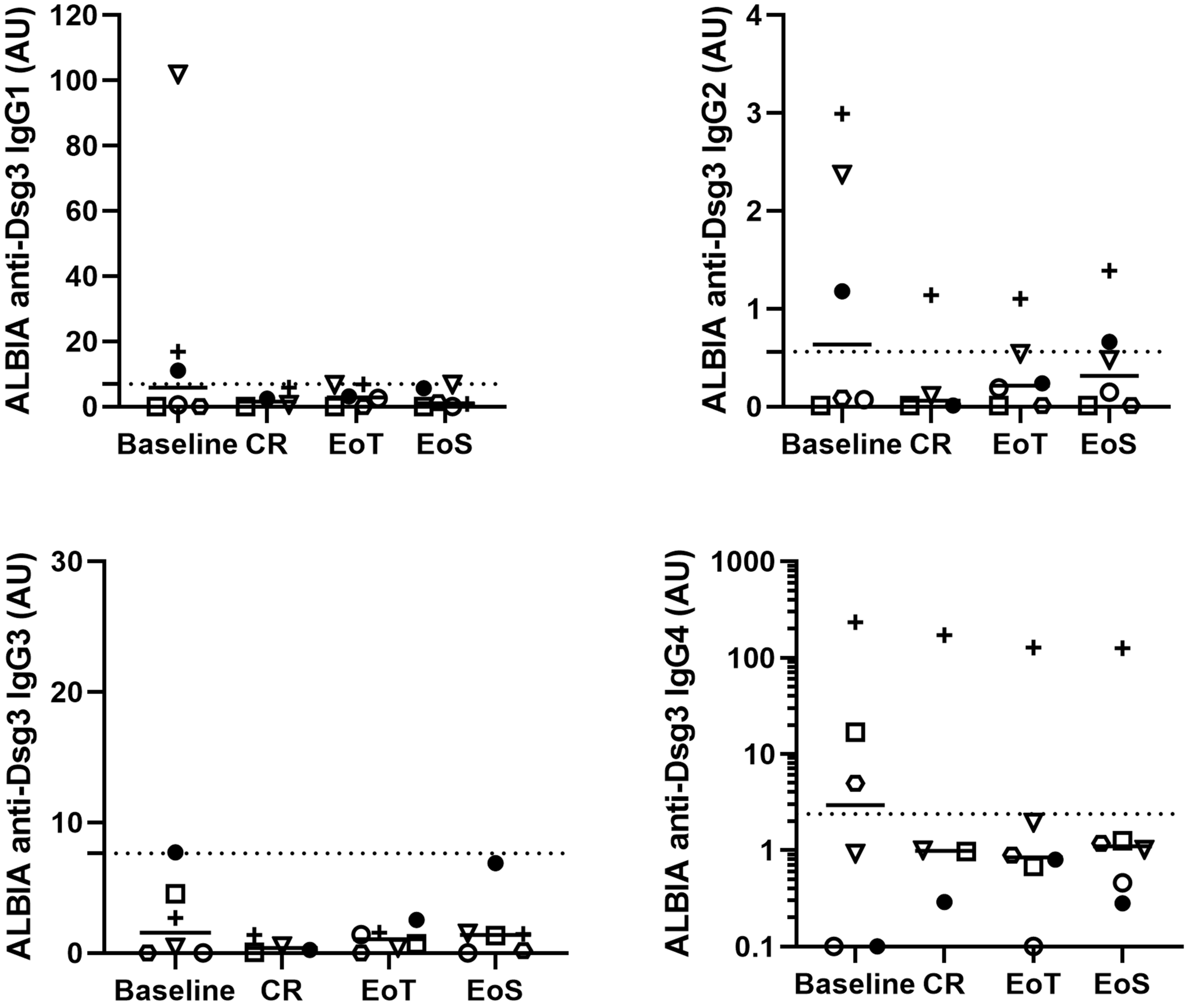

B

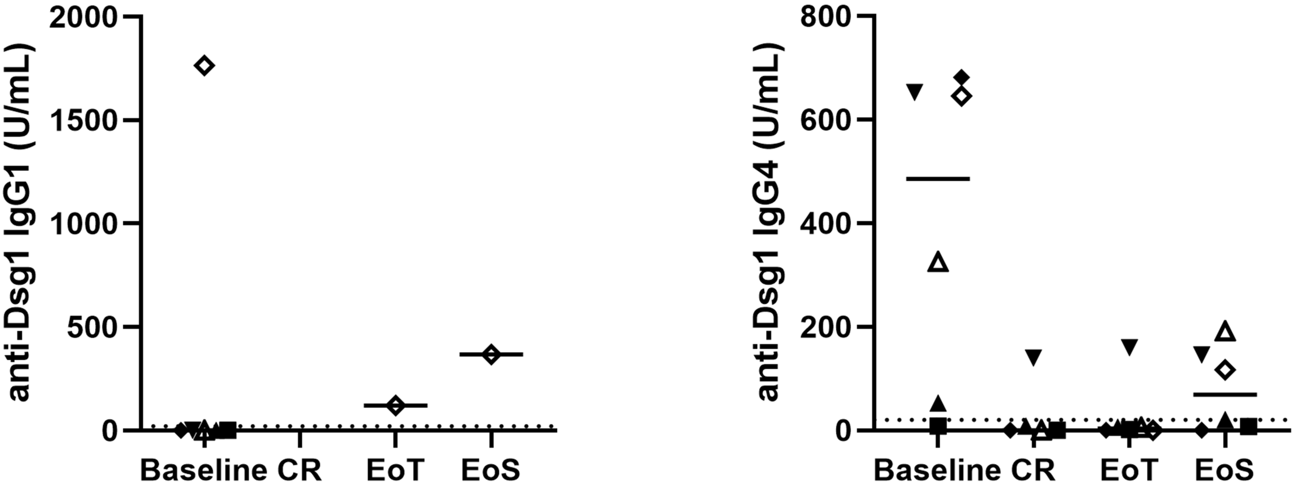

Supplemental Figure 3.

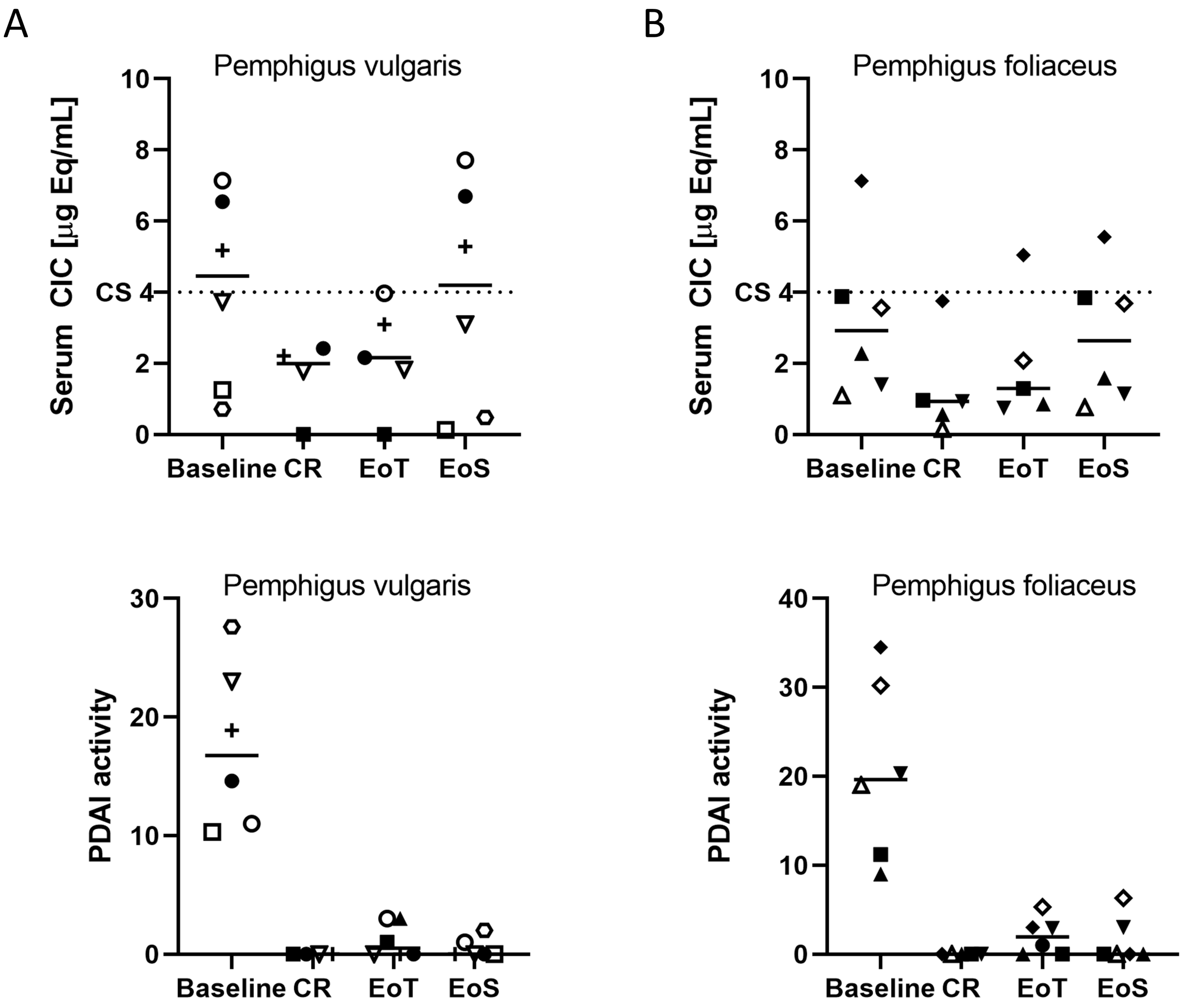

Supplemental Figure 4.

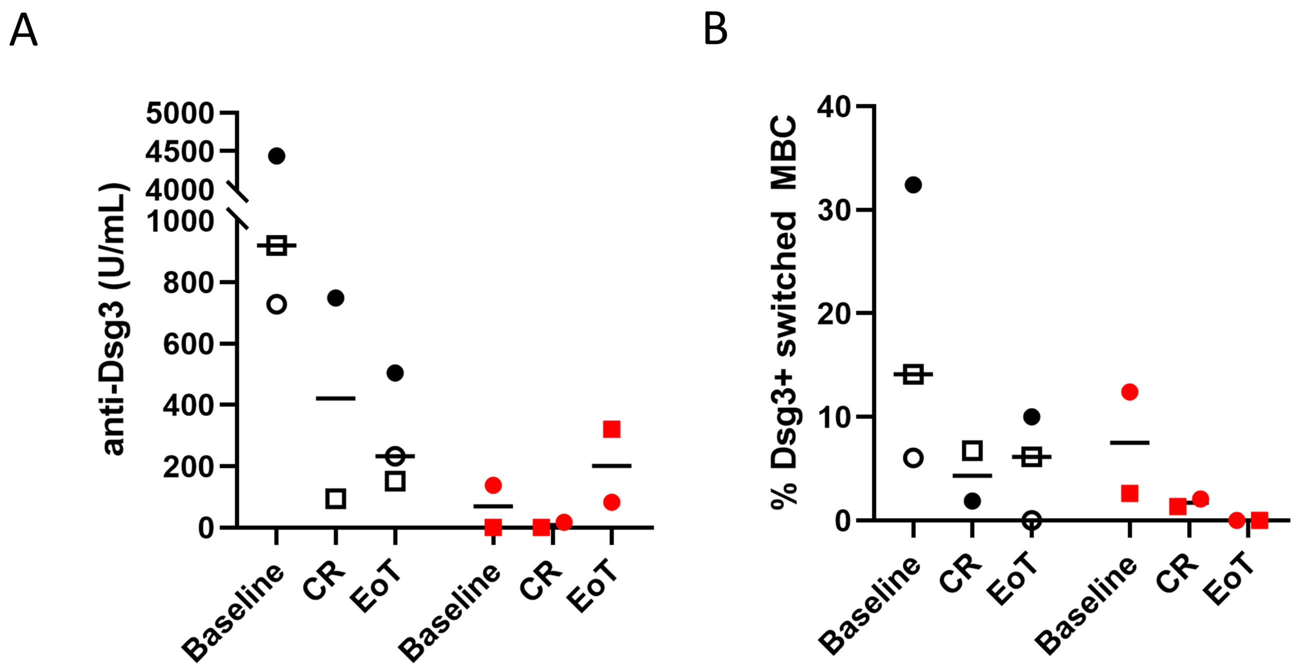

PV with Sustained Clinical Response (n=3), **black symbols**

PV with Relapse following CR (n=2), **red symbols**

Supplemental Figure 5.

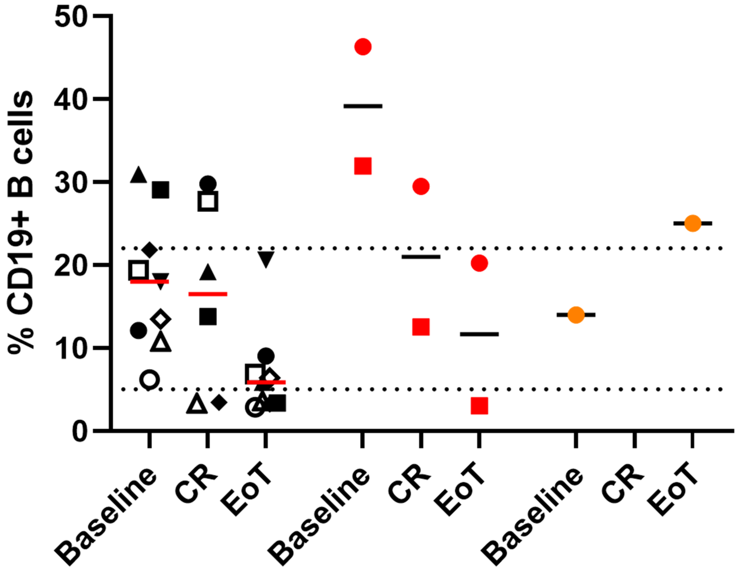

Sustained Clinical Response (n=9), **black symbols**

No clinical response (n=1), **orange symbols**

Relapsing participants (n=2), **red symbols**

Supplemental Table 1. Summary of individual clinical and serological responses by participants from cohort 3 and 4 who achieved sustained clinical response

		**PDAI activity**			**Anti-Dsg-1 titer (U/mL)**			**Anti-Dsg-3 titer (U/mL)**		
**Participant**	Diagnosis	BL	EoT	EoS	BL	EoT	EoS	BL	EoT	EoS
**1**	PF	9	0	0	75	12	29	-	-	-
**2**	PV	27.6	3	2	79	ND	ND	380	168	316
**3**	PF	20.3	2.9	3	252	40	76	-	-	-
**4**	PF	34.5	3	0	444	ND	ND	-	-	-
**5**	PV	14.6	0	0	18	ND	ND	4440*	504	632
**6**	PV	11	3	1	-	-	-	728	232	276
**7**	PF	11.2	0	0	62	29	29	-	-	-
**8**	PV	10.3	1	0	69	ND	ND	920*	152	380
**9**	PF	19	1	0	700	115	652	52	ND	ND
**10**	PF	30.2	5.3	6.3	660	112	127	-	-	-
**16**	PV	2	1	1	-	-	-	171	103	84
**17**	PV	3	1	1	59	19	11	392	444	272
**18**	PV	12	1	2.6	-	-	-	-	-	-
**20**	PV	23	0	0	129	ND	ND	2208*	63	272
**21**	PV	18.9	0	0	252	151	80	1738*	452	412
**22**	PV	14	0	0	9	9	20	580	97	328

BL, baseline; EoT, end of treatment; EoS, end of study; ND, non-detectable; PDAI, pemphigus disease area index; PV, pemphigus vulgaris; PF, pemphigus foliaceus. *values determined in the research laboratory on leftover serum samples. During the trial 800 U/mL was upper limit of quantification.
